# Are circulating microRNAs suitable for the early detection of malignant mesothelioma? Results from a nested case–control study

**DOI:** 10.1186/s13104-019-4113-7

**Published:** 2019-02-11

**Authors:** Daniel Gilbert Weber, Alexander Brik, Swaantje Casjens, Katarzyna Burek, Martin Lehnert, Beate Pesch, Dirk Taeger, Thomas Brüning, Georg Johnen, the MoMar study group, Bettina Dumont, Jan Gleichenhagen, Olaf Hagemeyer, Heike Heimann, Evelyn Heinze, Monika Kobek, Claudia Lechtenfeld, Swetlana Meier, Carmen Meinig, Simone Naumann, Simone Putzke, Irina Raiko, Hans-Peter Rihs, Peter Rozynek, Sandra Schonefeld, Katja Szafranski, Katharina Wichert, Thorsten Wiethege, Sandra Zilch-Schöneweis

**Affiliations:** Institute for Prevention and Occupational Medicine of the German Social Accident Insurance, Institute of the Ruhr University Bochum (IPA), Buerkle-de-la-Camp-Platz 1, 44789 Bochum, Germany

**Keywords:** Biomarker, Blood, Cancer, Liquid biopsies, Mesothelioma, MicroRNA, Marker, Negative results, Null results, Prediagnostic

## Abstract

**Objective:**

Malignant mesothelioma is an aggressive cancer of the serous membranes. For the detection of the tumor at early stages non- or minimally-invasive biomarkers are needed. The circulating biomarkers miR-132-3p, miR-126-3p, and miR-103a-3p were analyzed in a nested case–control study using plasma samples from 17 prediagnostic mesothelioma cases and 34 matched asbestos-exposed controls without a malignant disease.

**Results:**

Using prediagnostic plasma samples collected in median 8.9 months prior the clinical diagnosis miR-132-3p, miR-126-3p, and miR-103a-3p revealed 0% sensitivity on a defined specificity of 98%. Thus, the analyzed miRNAs failed to detect the cancer in prediagnostic samples, showing that they are not feasible for the early detection of malignant mesothelioma. However, the miRNAs might still serve as possible markers for prognosis and response to therapy, but this needs to be analyzed in appropriate studies.

**Electronic supplementary material:**

The online version of this article (10.1186/s13104-019-4113-7) contains supplementary material, which is available to authorized users.

## Introduction

Malignant mesothelioma is an asbestos-related malignancy with increasing incidence worldwide [1]. Based on the continued use of asbestos and a long latency period of up to 50 years mesothelioma remains a global health problem. Commonly, symptoms occur at late stages of the disease and median survival after diagnosis is between nine and 13 months, depending on treatment [2]. A diagnosis of mesothelioma at early stages, when the tumor is still small and has not spread, might be a promising opportunity to improve therapy options. Current therapies are improved in rather small steps [3] and promising approaches like immunotherapy are being investigated [4]. Notably, Jones et al. presented a case with an exceptional and sustained response to immune checkpoint inhibition [5]. In order to finally reduce mortality there is a need to identify and validate appropriate biomarkers for the early detection of cancer, particularly for mesothelioma [6].

To detect cancer at early stages, non- and minimally-invasive methods like liquid biopsies are preferable. Circulating miRNAs are well-known biomarkers of several diseases including cancer and might be feasible for early detection [7], e.g., liver cancer was detected prior to a common clinical diagnosis using circulating miRNAs [8, 9]. For the detection of malignant mesothelioma miR-16 [10], miR-17 [10], miR-30e-3p [11], miR-103a-3p [12], miR-126 [13], miR-625-3p [14], miR-132-3p [15], and miR-486 [10] were introduced as blood-based biomarkers. However, the miRNAs in those studies were analyzed using cross-sectional designs, commonly resulted in study groups with higher numbers of cancer patients at late stages. Thus, the biomarkers do not allow to draw any conclusion regarding their performance for the detection of mesothelioma at early stages [16]. Most of the introduced biomarker candidates are not validated in samples collected prior to a diagnosis of malignant mesothelioma. Hence, longitudinal studies, which facilitate repeated sampling of an at-risk population, are important for the evaluation of the biomarker performance for the early detection of the cancer.

The aim of this study was to analyze circulating miRNAs in a case–control study nested into a prospective cohort to assess the biomarker performance for the detection of malignant mesothelioma in prediagnostic plasma samples.

## Main text

### Methods

The MoMar (Molecular Markers) cohort consists of 2769 German workers formerly exposed to asbestos with a confirmed asbestos-related occupational disease (asbestosis and/or other (nonmalignant) pleural diseases caused by asbestos). The participants were recruited from November 2008 to February 2018 at 26 medical centers in Germany. Voluntary blood donation and a questionnaire were offered every year [17]. Using a nested case–control design, 17 male mesothelioma patients including ten epithelioid (58.8%), two biphasic (11.8%), and three sarcomatoid (17.6%) mesotheliomas were investigated. In two cases (11.8%) the histological subtype of the tumor remained unknown. The median time between blood collection and date of diagnosis was 8.9 months. To each case two cancer-free controls were matched. Thus, the control group comprised 34 men of the MoMar cohort. Criteria for matching were gender, age, smoking status, and date of blood collection. Characteristics of the study groups are presented in Additional file 1 and detailed characteristics of the subjects in Additional file 2.

Peripheral blood was collected in 9.0 ml S-Monovette EDTA gel tubes (Sarstedt, Nümbrecht, Germany). Within 30 min after blood collection samples were centrifuged at 2000×*g* for 10 min at room temperature. After centrifugation plasma was separated from the cellular fraction and both matrices were immediately frozen and temporarily stored in the collaborating study centers. Samples were regularly picked up, transported to the central laboratory, aliquoted using an automated liquid handling robot (Tecan Group Ltd., Männedorf, Switzerland), and stored at − 80 °C until use. For the determination of miR-132-3p and miR-126-3p, RNA from 0.5 ml plasma was isolated using the miRVana PARIS kit (Thermo Fisher Scientific, Darmstadt, Germany) according to the manufacturer's instructions, modified by adding 5 µl Carrier RNA MS2 (Roche, Mannheim, Germany). For the determination of miR-103a-3p, RNA from 0.5 ml cellular fraction was isolated using the RiboPure-Blood Kit according to the Alternate protocol: Isolation of Small RNAs (Thermo Fisher Scientific). Individual miRNAs were analyzed using commercial TaqMan microRNA assays (Life Technologies) for miR-103a-3p (ID 000439), miR-125a (ID 000448), miR-132-3p (ID 000457), miR-146b-5p (ID 001097), miR-126-3p (ID 002228), and U6 snRNA (ID 001973) as described previously [15]. Quantitative miRNA expression data were acquired using the ABI SDS software (Thermo Fisher Scientific) and presented in Additional file 2. Estimation of the cycle threshold (Ct) was performed as described elsewhere [12, 15]. Normalization was performed with the 2^−∆Ct^ method [18] using miR-146b-5p, U6 snRNA, and miR-125a, as references for miR-132-3p, miR-126-3p, and miR-103a-3p, respectively.

Box plots with median and inter-quartile range (IQR) were used to depict the distribution of single biomarkers and their combination. Whiskers represent minimum and maximum values. Mann–Whitney U tests or Kruskal–Wallis tests were applied to examine group differences and p-values < 0.05 were considered as statistically significant. Receiver operating characteristic (ROC) curves were used to quantify classification performance of the biomarkers. The accuracy of the diagnostic tests was depicted by the area under curve (AUC) and its 95% confidence interval (CI). The ROC curves of the biomarker combination were calculated with each miRNA as independent variable in a multiple logistic regression model. Sensitivities and specificities were determined using a defined specificity of 98% or maximum Youden’s Index (YI). All statistical analyses were performed using SAS/STAT and SAS/IML software, version 9.4 (SAS Institute Inc., Cary, USA). Graphs were generated using GraphPad Prism (GraphPad Software, La Jolla, USA).

### Results

**Fig. 1:**
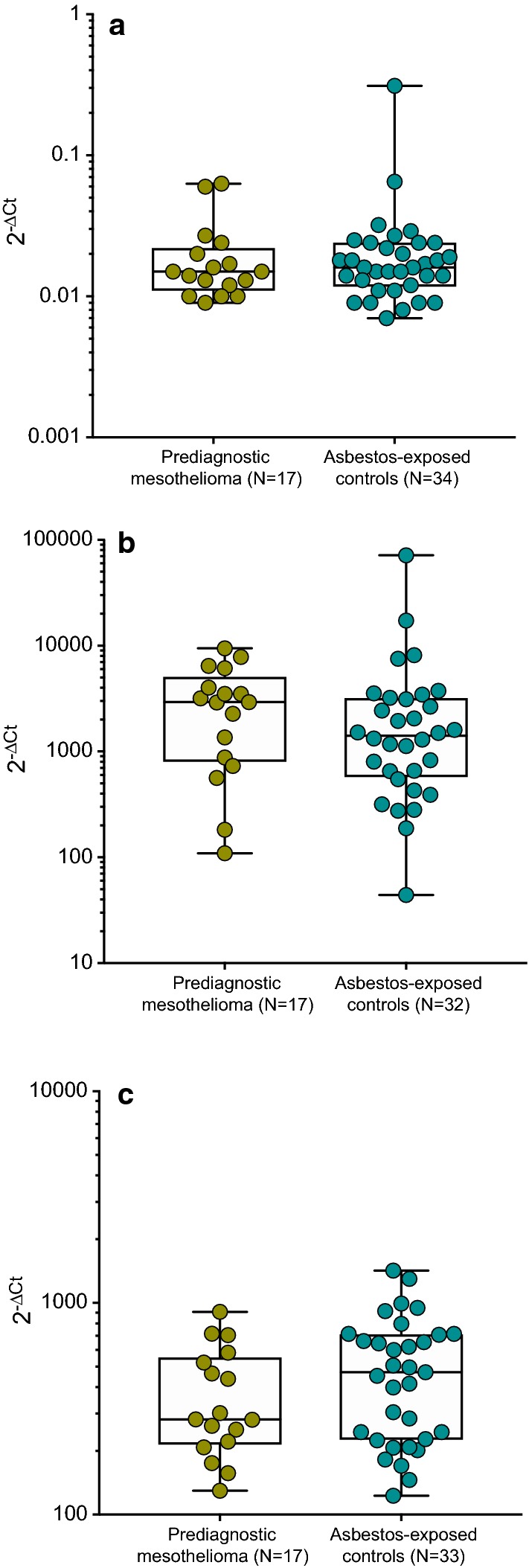
Distribution of miR-132-3p (**a**), miR-126-3p (**b**), and miR-103a-3p (**c**) in prediagnostic mesothelioma cases and asbestos-exposed controls. Mann–Whitney U tests were performed to examine group differences. Horizontal bars represent median and interquartile range

The two miRNAs miR-132-3p and miR-126-3p were determined in prediagnostic plasma samples. The median level of miR-132-3p was 0.015 (IQR 0.012–0.020) for mesothelioma cases and 0.016 (IQR 0.012–0.024) in the control group (Fig. 1a). The median level of miR-126-3p was 2925 (IQR 878–4012) in the mesothelioma group and 1413 (IQR 600–3170) for cancer-free controls (Fig. 1b). In two control subjects miR-126-3p could not be determined. The miRNA miR-103a-3p was measured in the cellular fraction of prediagnostic blood samples. The median level of miR-103a-3p was 282 (IQR 221–523) in mesothelioma cases and 471 (IQR 228–707) in the control group (Fig. 1c). In one control subject miR-103a-3p could not be determined. Group differences were not statistically significant for any tested miRNA. Additionally, prediagnostic mesothelioma cases were divided in three subgroups according to the time between blood collection and diagnosis [< 6 months (N = 5), 6–12 months (N = 7), and ≥ 12 months (N = 5)]. No statistically significant differences between the groups could be observed for any analyzed miRNA (Additional file 3).

**Fig. 2:**
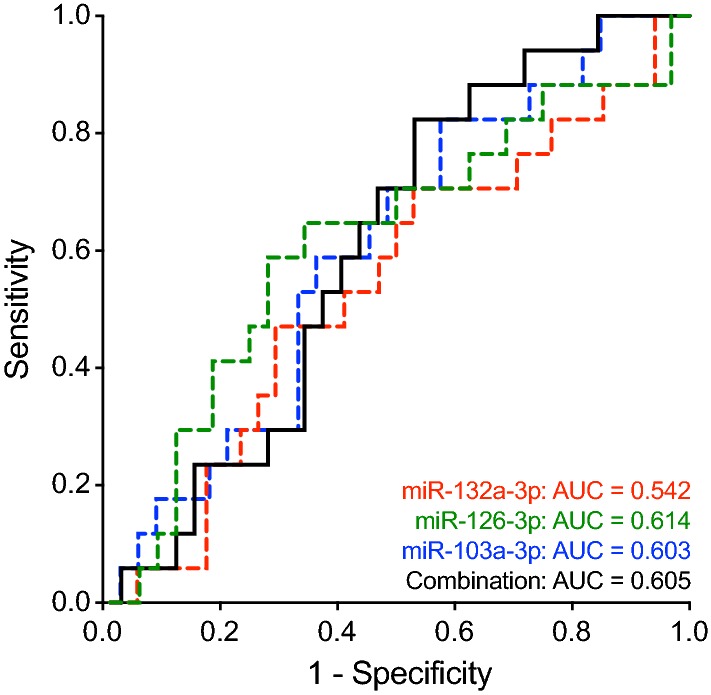
Receiver operating characteristic (ROC) curves of miR-132-3p, miR-126-3p, miR-103a-3p, and the combination of the three miRNAs

The ROC analyses showed an AUC of 0.542 (95% CI 0.370–0.713) for miR-132a-3p, 0.614 (95% CI 0.439–0.789) for miR-126-3p, and 0.603 (95% CI 0.440–0.765) for miR-103a-3p (Fig. 2). Using a defined specificity of 98%, a sensitivity of 0% was revealed for each miRNA. Using maximum YI resulted in 71%, 59%, and 82% sensitivity and 47%, 72%, and 42% specificity for miR-132-3p, miR-126-3p, and miR-103a-3p, respectively (Table 1). The combination of all three miRNAs revealed an AUC of 0.605 (95% CI 0.445–0.765). The combined ROC curve is displayed in Fig. 2. Using a defined specificity of 98% resulted in 0% sensitivity and using maximum YI resulted in 82% sensitivity and 47% specificity for the biomarker combination (Table 1).

**Table 1:** Sensitivities and specificities of miR-132a-3p, miR-126-3p, miR-103a-3p, and the combination of the three miRNAs

Biomarker	Sensitivity (%)	Specificity (%)
miR-132-3p		
Defined specificity	0	98
Maximum YI	71	47
miR-126-3p		
Defined specificity	0	98
Maximum YI	59	72
miR-103a-3p		
Defined specificity	0	98
Maximum YI	82	42
Combination of the three miRNAs		
Defined specificity	0	98
Maximum YI	82	47

### Discussion

For cancer detection in prediagnostic samples, high specificities of the biomarkers are needed to receive a preferable low number of false-positive tests in order to avoid psychological distress and unnecessary invasive examinations for the patients [16]. Using a defined specificity of 98% none of the mesothelioma cases were detected by miR-132-3p and miR-126-3p in plasma, as well as miR-103a-3p in the cellular fraction of blood. Similarly, using maximum YI resulted in low sensitivities and specificities. Also, the combination of the three biomarkers did not improve the marker performance. Thus, the analyzed candidate biomarkers failed to detect malignant mesothelioma in prediagnostic blood samples, showing that they are not feasible for the early detection of this cancer. Originally, for miR-132-3p, miR-126-3p, and miR-103a-3p promising sensitivities of 86%, 61%, and 83%, and specificities of 73%, 74%, and 71%, respectively, were revealed for the discrimination of mesothelioma patients and asbestos-exposed controls [12, 13, 15]. However, compared to the results obtained with already manifest tumors, the performance of the analyzed miRNAs dropped substantial using prediagnostic samples.

Globally, miRNAs are down-regulated in cancer patients [19] and the same trend could be observed for miR-132-3p, miR-126-3p, and miR-103a-3p in manifest mesothelioma [12, 13, 15]. However, the down-regulation of circulating miRNAs is not a result of a down-regulation of these miRNAs within the tumor [20]. The tumor growth might rather negatively affect the miRNA expression in other body cells releasing the miRNAs. Thus, the down-regulation of circulating miRNAs might be a non-specific response to the presence of neoplastic growth [20]. It remains plausible that the tumor needs to have a sufficient size for a distinct effect on the miRNA expression in other cells. The analyzed blood samples in this study were taken from prediagnostic mesothelioma patients on average 8.9 months prior to the clinical diagnosis based on symptoms or a health impairment. Thus, the tumor size might be comparatively small at the time of blood collection. It is comprehensible that the expression of the circulating miRNAs is not yet down-regulated in prediagnostic cancer samples in contrast to the initial identification studies using samples from manifest mesothelioma cases [12, 13, 15].

However, circulating miRNAs are described as generally suitable to predict prognosis and response to therapy [21] and this might be also true for miR-132-3p, miR-126-3p, and miR-103a-3p. Thus, appropriate studies have to be carried out to assess the performance of the miRNAs for these applications.

For the detection of cancer at early stages, the use of circulating biomarkers in prediagnostic samples remains to be a meaningful approach, because biomarkers are economical, easy to apply, and might be implemented in clinical routine without much effort. Blyuss et al. demonstrated that the combination of CA125, HE4, and glycodelin detected ovarian cancer in blood samples up to 1 year before the clinical diagnosis with a sensitivity and specificity > 90% [22]. For malignant mesothelioma, Johnen et al. showed that the combination of mesothelin and calretinin detected the tumor using prediagnostic plasma samples up to 15 months prior the clinical diagnosis with a sensitivity of 46% at a defined specificity of 98% [17]. In contrast to the analyzed circulating miRNAs, the above-mentioned proteins might be released directly from the tumor, resulting in a sustainable detection even at early stages of the disease. The combination of several biomarkers within a panel improves the diagnostic performance [17, 22–25]. Thus, it is still important to identify and validate more biomarkers of different molecular classes, i.e. proteins as well as miRNAs, circular RNAs, long non-coding RNAs, and DNA-Methylation, for the detection of mesothelioma at early stages.

## Limitations

Generally, in studies with a longitudinal design the number of incident cases of mesothelioma during follow-up is low. At the time point of the realized analyses only 17 samples from prediagnostic cancer cases that were diagnosed during a period of about 9 years were included.

Only three miRNAs were analyzed in this study, although miR-16, miR-17, and miR-486 [10], miR-30e-3p [11], and miR-625-3p [14] were also suggested as blood-based biomarkers for mesothelioma. However, miR-16, miR-17, and miR-486 seem to be affected by hemolysis [26–28] making them not appropriate as blood-based biomarker in clinical routine. MiR-30e-3p were described as candidate biomarkers using extracellular vesicles. Unfortunately, the isolation of vesicles from plasma samples was not part of this study. MiR-625-3p was not analyzed in this study, because the initially described biomarker performance could not be verified in an independent study [15].

## Additional files

**Additional file 1.** Characteristics of prediagnostic mesothelioma cases and asbestos-exposed controls in the nested case–control study.

**Additional file 2.** Detailed characteristics of the study subjects and quantitative miRNA expression data.

**Additional file 3.** Distribution of miR-132-3p (A), miR-126-3p (B), and miR-103a-3p (C) in prediagnostic mesothelioma cases, categorized by time between blood collection and diagnosis (< 6 months, 6–12 months, and ≥ 12 months). Kruskal–Wallis tests were performed to examine group differences. Horizontal bars represent median and interquartile range.

## Abbreviations

AUC: area under curve
CI: confidence interval
Ct: cycle threshold
IQR: interquartile range
miRNA: microRNA
ROC: receiver operating characteristics
YI: Youden’s Index
